# Proteomes of primary skin fibroblasts from healthy individuals reveal altered cell responses across the life span

**DOI:** 10.1111/acel.13609

**Published:** 2022-04-15

**Authors:** Dimitrios Tsitsipatis, Jennifer L. Martindale, Ceereena Ubaida‐Mohien, Alexey Lyashkov, Hagai Yanai, Amogh Kashyap, Chang Hoon Shin, Allison B. Herman, Eunbyul Ji, Jen‐Hao Yang, Rachel Munk, Christopher Dunn, Yevgeniya Lukyanenko, Xiaoling Yang, Chee W. Chia, Ajoy C. Karikkineth, Linda Zukley, Jarod D’Agostino, Mary Kaileh, Chang‐Yi Cui, Isabel Beerman, Luigi Ferrucci, Myriam Gorospe

**Affiliations:** ^1^ Laboratory of Genetics and Genomics National Institute on Aging National Institutes of Health Intramural Research Program Baltimore Maryland USA; ^2^ Translational Gerontology Branch National Institute on Aging National Institutes of Health Intramural Research Program Baltimore Maryland USA; ^3^ Laboratory of Molecular Biology and Immunology National Institute on Aging National Institutes of Health Intramural Research Program Baltimore Maryland USA; ^4^ Clinical Research Core National Institute on Aging National Institutes of Health Intramural Research Program Baltimore Maryland USA

**Keywords:** aging, autophagy, DNA damage, DNA repair, human dermal fibroblasts, proteomics, reactive oxygen species, ribosome biogenesis

## Abstract

Changes in the proteome of different human tissues with advancing age are poorly characterized. Here, we studied the proteins present in primary skin fibroblasts collected from 82 healthy individuals across a wide age spectrum (22–89 years old) who participated in the GESTALT (Genetic and Epigenetic Signatures of Translational Aging Laboratory Testing) study of the National Institute on Aging, NIH. Proteins were extracted from lysed fibroblasts and subjected to liquid chromatography‐mass spectrometry analysis, and the expression levels of 9341 proteins were analyzed using linear regression models. We identified key pathways associated with skin fibroblast aging, including autophagy, scavenging of reactive oxygen species (ROS), ribosome biogenesis, DNA replication, and DNA repair. Changes in these prominent pathways were corroborated using molecular and cell culture approaches. Our study establishes a framework of the global proteome governing skin fibroblast aging and points to possible biomarkers and therapeutic targets.

## Graphical abstract

The proteins expressed in different cell types with advancing human age are poorly characterized. Here, we used mass spectrometry to study the proteins present in primary skin fibroblasts collected from 82 healthy individuals across a wide age spectrum. We identified and validated key pathways changing with skin fibroblast aging, including autophagy, scavenging of reactive oxygen species, ribosome biogenesis, and DNA replication and repair.

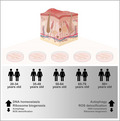

**Abbreviations**
ATG: autophagy related
B2M: beta‐2‐microglobulin
BECN1: beclin‐1
BGAL: β‐galactosidase
BMI: body mass index
BrdU: bromodeoxyuridine
CADM1: cell adhesion molecule 1
CSPG4: chondroitin sulfate proteoglycan 4
CTGF/CCN2: connective tissue growth factor/cellular communication network factor 2
ECM: extracellular matrix
GABARAP: GABA type A receptor‐associated protein
GAPDH: glyceraldehyde‐3‐phosphate dehydrogenase
GESTALT: Genetic and Epigenetic Signatures of Translational Aging Laboratory Testing
GLRX1: glutaredoxin 1
GSTK1: glutathione S‐transferase kappa 1
HDF: human dermal fibroblasts
IF4G2: eukaryotic translation initiation factor 4 gamma 2
ITB5: Integrin subunit beta 5
LAMP2: lysosome‐associated membrane protein 2
LC3: microtubule‐associated protein 1A/1B‐light chain 3
LC‐MS: liquid chromatography‐mass spectrometry
MCM: minichromosome maintenance
MCM6: minichromosome maintenance complex component 6
MGST1: microsomal glutathione S‐transferase 1
mTOR: mechanistic target of rapamycin; SE, short exposure
mTORC1: mTOR kinase complex
PIK3C3: class III phosphatidylinositol 3‐kinase
PLS: partial least squares
PTGIS: prostaglandin I2 synthase
PURA: PUR element‐binding protein α
ROS: reactive oxygen species
RT‐qPCR: reverse transcription quantitative PCR
SOD1: CuZn‐superoxide dismutase
SQSTM1: sequestosome‐1
TGF: transforming growth factor
TMT: tandem mass tag
TRXR1: thioredoxin, reductase 1
WDFY3: WD repeat and FYVE domain‐containing protein 3
WIPI2: WD repeat domain phosphoinositide‐interacting 2

## 1 INTRODUCTION

One of the most evident phenotypes of advancing age is skin aging. Driven by intrinsic factors (genes) and extrinsic factors (environmental exposures), skin aging is important because it can compromise the outer barrier that protects internal organs (Farage et al., 2008; Zhang & Duan, 2018). Skin is comprised of the ectoderm‐derived thin epidermis, the mesoderm‐derived thick dermis, and the hypodermis. All skin layers show characteristic changes with age, but dermal atrophy is the major contributor to wrinkle formation and to the thinning and sagging of skin during aging. The skin dermis mainly consists of fibroblasts, which produce the components of the extracellular matrix (ECM), including collagens, fibronectins, elastin, and glycoproteins. In dermal aging, fibroblasts decline both in number and in capacity of biosynthesis of ECM, contributing to dermal atrophy and loss of ability to remodel the ECM (Braverman, 2000; Makrantonaki & Zouboulis, 2007; Shuster et al., 1975). At a molecular level, reduced signaling through transforming growth factor‐beta (TGF‐β, TGFB1), SMAD proteins, and connective tissue growth factor/cellular communication network factor 2 (CTGF/CCN2) contributes to the marked reduction in collagens in aged skin (Lovell et al., 1987; Quan et al., 2010). The loss of skin integrity and rise in permeability increase the risk of damage and infection of internal organs.

Given the importance of maintaining an intact barrier for the body, it is critical to understand the molecular mechanisms that contribute to skin aging. Here, we set out to investigate the proteome of skin fibroblasts in healthy individuals dispersed over a wide age range. Punch biopsies were obtained from 82 participants who were evaluated as healthy according to stringent clinical and functional criteria, spanning a wide age range—22 to 89 years old—from the Genetic and Epigenetic Signatures of Translational Aging Laboratory Testing (GESTALT) study of the National Institute on Aging (NIA), National Institutes of Health (NIH) (Tanaka et al., 2018; Tumasian et al., 2021; Ubaida‐Mohien et al., 2019). Our study builds upon earlier reports of proteomes in cultured skin fibroblasts collected from smaller subsets of individuals (Boraldi et al., 2003; Waldera‐Lupa et al., 2014). Biopsies were obtained from the inner axilla, which is typically not exposed to sunlight (Fisher et al., 2002; McCabe et al., 2020). Following deep quantitative proteomic analysis, we found numerous proteins differentially expressed as a function of donor age. These proteins govern prominent pathways that change with age, specifically autophagy, antioxidant defense, ribosome biogenesis, and DNA replication and repair. The proteins and pathways identified in this study warrant attention in the design of therapeutic interventions for skin aging.

## 2 RESULTS

### 2.1 Proteomic analysis of cultured fibroblasts from skin biopsies of GESTALT donors aged 22 to 89 years old

**FIGURE 1:**
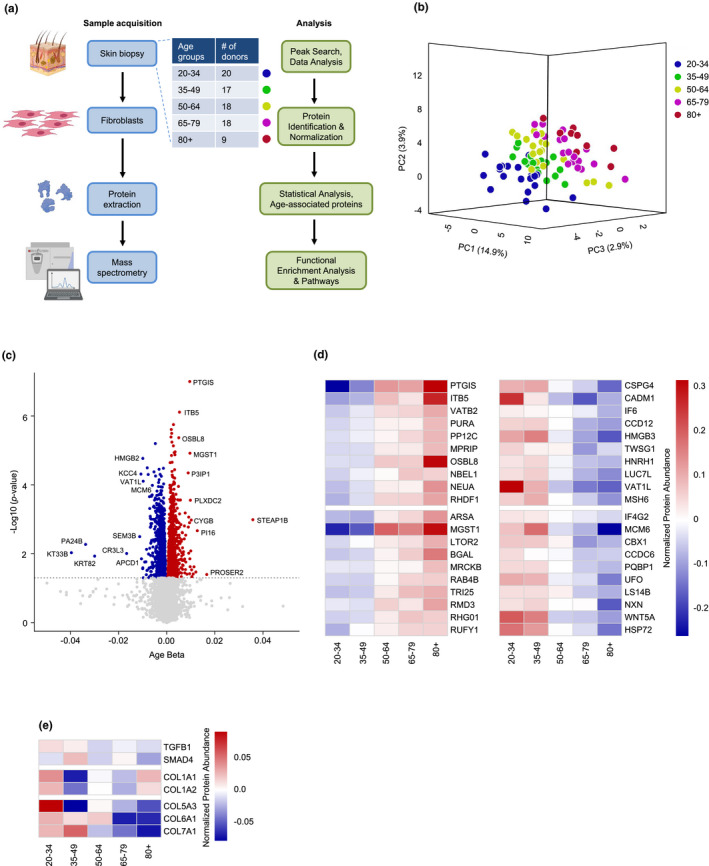
Initial screening of the HDF proteome. (a) Workflow of sample acquisition and preparation (blue) and bioinformatic analysis (green) followed in this study. Classification of different age groups and number of donors per group (table). (b) PLS analysis of age‐associated proteins from five age groups: 20–34, 35–49, 50–64, 65–79, and 80+ years old. (c) Volcano plot showing beta coefficients of proteins regressed with age (per year). Proteins showing significantly increased (red dots) or decreased (blue dots) levels with age (*p* < 0.05, adjusted for covariates). Gray dots show proteins that did not change significantly with age (*p* > 0.05). (d) Heatmaps of the top 20 significantly elevated proteins (left) and top 20 significantly reduced proteins (right) with age based on *p*‐adjusted values. (e) Heatmap of the levels of prominent proteins governing the expression of collagens (TGFB1 and SMAD4) and different collagens (COL1A1, COL1A2, COL5A3, COL6A1, and COL7A1) across the age groups

Primary human diploid fibroblasts (HDFs) were obtained from skin biopsies collected from 82 individual donors in the GESTALT cohort (NIA, NIH); each of the 82 fibroblast cultures was established and expanded for analysis. Total protein was then extracted from the cells and subjected to mass spectrometry analysis (Figure 1a and Supplementary File S1). Briefly, fibroblast protein samples were digested into peptides using an in‐solution trypsin digestion protocol, and TMT10plex labeling was performed on peptide samples in 9 batches, each containing one reference sample for normalization across runs. TMT‐labeled peptide samples were fractionated and pooled into 15 fractions each before analysis on an Orbitrap Fusion Lumos Mass Spec instrument. To avoid bias, donor IDs were blinded, and TMT channels were randomized between runs. Quantification of proteins and peptides was based on TMT reporter ion intensities. The mass spectrometry proteomic data are deposited (see Data Availability). As indicated by three‐dimensional partial least squares (PLS) analysis, individuals within the same age group tended to cluster together and there was clear separation among the five age groups (Figure 1b). In total, 9,341 proteins were quantified, 2,072 proteins were associated with age with a statistical significance of *p *< 0.05, and among them, 268 proteins were still significantly associated with aging after Benjamini–Hochberg correction *p* < 0.05 (Figure 1c). Of these, most proteins showed higher abundance with older age, like prostaglandin I2 synthase (PTGIS), integrin subunit beta 5 (ITB5), PUR element‐binding protein α (PURA), microsomal glutathione S‐transferase (MGST1), and β‐galactosidase (BGAL). Other proteins showed lower abundance with older age, such as chondroitin sulfate proteoglycan 4 (CSPG4), cell adhesion molecule 1 (CADM1), minichromosome maintenance complex component (MCM) 6, and eukaryotic translation initiation factor 4 gamma 2 (IF4G2) (Figure 1d).

Given that collages are typically reduced in aging skin (El‐Domyati et al., 2002; Lovell et al., 1987), we examined the levels of collagens in our proteomic datasets as an internal test of our analysis. We first focused on a pathway of collagen biosynthesis involving TGF‐β and SMAD4, two proteins that were modestly underrepresented with age (Figure 1e). In line with these changes, the levels of type I collagen (COL1A1 and COL1A2), the most abundant collagen, were also reduced across the age groups through the 65–79 age group (Figure 1e), although the levels of COL1A1 were modestly elevated in the ≥80‐year‐old group. Other collagens, including COL5A3, COL6A1, and COL7A1, which are less abundant, were also underrepresented with age. Taken together, and in line with previous reports (El‐Domyati et al., 2002), the levels of many collagen proteins were modestly lower with age in this healthy cohort (Figure 1e).

To better understand whether the collective changes in proteins affected cell functions in skin fibroblasts from older individuals, we identified differentially represented protein pathways across the age groups using unsupervised clustering, and examined the levels of prominent proteins in these pathways by linear regression analysis (Supplementary File S1). This analysis revealed several pathways more active (section 2.2) and several less active (section 2.3) as a function of donor age.

### 2.2 Increased autophagy and ROS detoxification pathways in skin fibroblasts from older donors

**FIGURE 2:**
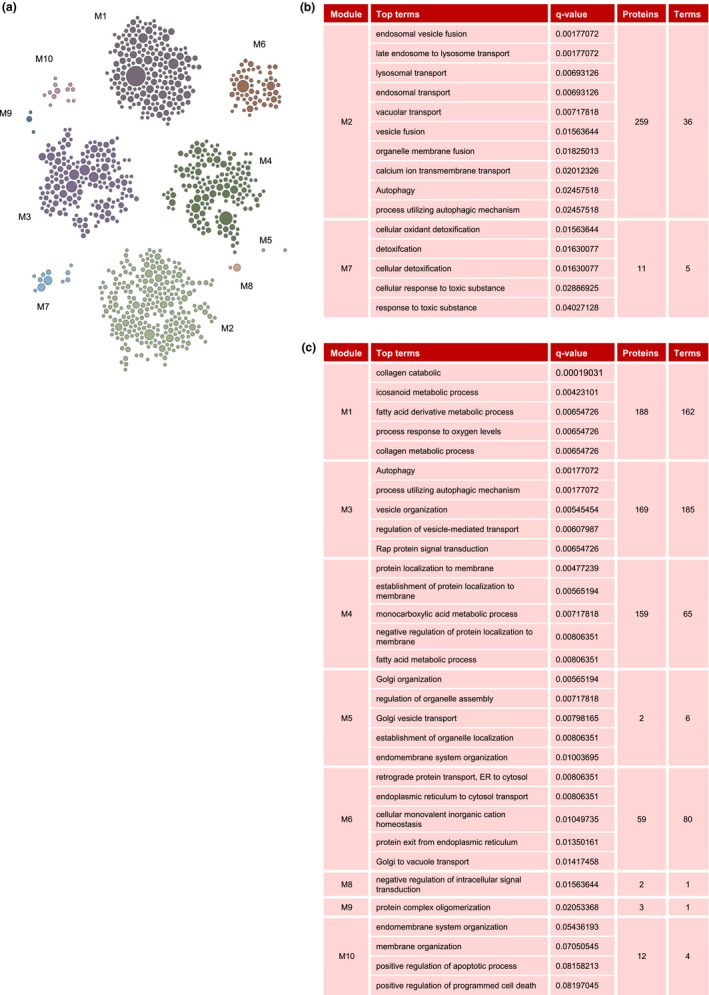
Pathways significantly overrepresented with age. (a) Modules significantly overrepresented with age. (b) Each module consists of significantly increased pathways (top term) with shared proteins. We further investigated modules 2 and 7 (M2 and M7), representing autophagy and detoxification/ROS scavenging, respectively. (c) Remaining modules overrepresented with older age

Using STRING pathway enrichment analysis tool and HumanBase functional module analysis, we identified cohesive gene clusters and process‐specific functional relationship networks involving proteins that were more abundant with age (Figure 2a). Among these functional modules, we focused on autophagy and ROS detoxification, represented in modules 2 and 7 of our proteomic analysis (Figure 2b); other key pathway modules also elevated with age are shown (Figure 2c).

**FIGURE 3:**
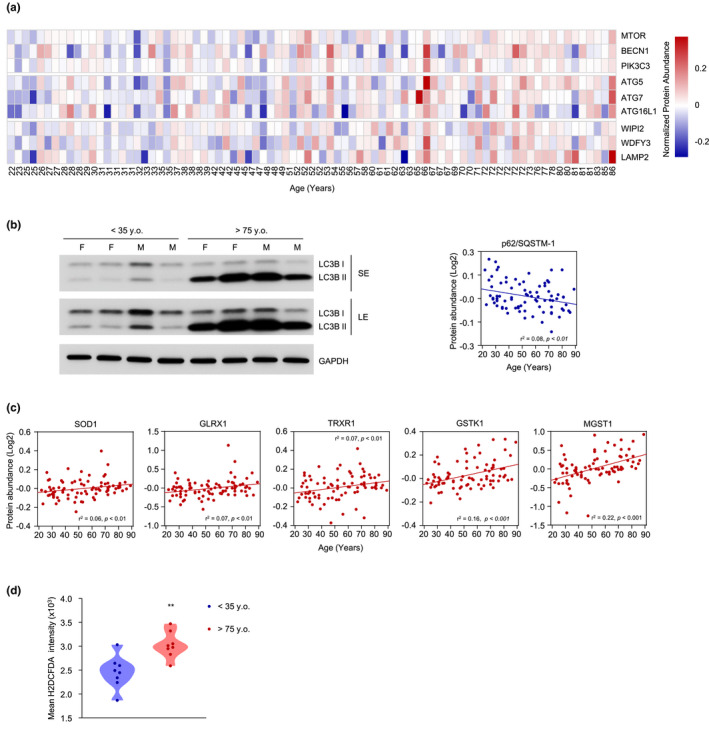
Higher levels of proteins implicated in autophagy and detoxification with age and assessment of the respective pathways. (a) Heatmap of autophagy‐related proteins in each of the 82 donors (columns). Donor ages are indicated at the bottom. (b) Western blot analysis of LC3‐I and LC3‐II in 4 donors <35 years old and 4 donors >75 years old, including both female (F) and male (M) donors. GAPDH levels were included to monitor loading (left). Graph, linear regression analysis of p62/SQSTM1 levels from the proteomic analysis (right). (c) Linear regression graphs of significantly overrepresented proteins associated with ROS scavenging. (d) Accumulation of ROS in 8 donors <35 years old and 8 donors >75 years old as assessed by flow cytometry (Materials and Methods). Mean intensity of FITC (H_2_DCFDA). BECN1, Beclin‐1; PIK3C3, class III phosphatidylinositol 3‐kinase; GLRX1, glutaredoxin 1; GSTK1, glutathione S‐transferase kappa 1; LAMP2, lysosome‐associated membrane protein 2; LE, long exposure; MGST1, microsomal glutathione S‐transferase; mTOR, mechanistic target of rapamycin; SE, short exposure; SOD1, CuZn‐superoxide dismutase, CuZn‐SOD; SQSTM1, p62/Sequestosome‐1; TRXR1, thioredoxin, reductase 1; WDFY3, WD repeat and FYVE domain‐containing protein 3; WIPI2, WD repeat domain phosphoinositide‐interacting 2. Significance was established using Student's *t* test. **p ≤ 0.01

A major pathway of protein turnover and recycling of cellular components, autophagy has been found to be dysregulated in aging and age‐related pathologies, whereas enhanced autophagy is associated with features of slower aging and longevity (Aman et al., 2021; Rubinsztein et al., 2011). In our proteomic analysis, we found overrepresentation with age of proteins ATG5, ATG7, and ATG16L1, three members of the ubiquitin‐like conjugation system involved in the expansion of autophagosomes and LC3 lipidation (Wesselborg & Stork, 2015) (Figure 3a). We also found that older donor age correlated with higher levels of Beclin‐1 (BECN1), which has a prominent role in promoting the nucleation step of autophagy (Figure 3a) (Funderburk, 2010). The levels of other positive regulators of autophagy, including WD repeat domain phosphoinositide‐interacting 2 (WIPI2), the lysosome‐associated membrane protein 2 (LAMP2), class III phosphatidylinositol 3‐kinase (PIK3C3/VPS34), and WD repeat and FYVE domain‐containing protein 3 (WDFY3), were also higher (Figure 3a). The rise in abundance of these proteins was interesting because PIK3C3 recruits prominent autophagy regulators, including WIPI2, which can positively regulate LC3 lipidation and thus contribute to the maturation of omegasomes to autophagosomes (Polson et al., 2010), while LAMP2 regulates chaperone‐mediated autophagy, and WDFY3 modulates the clearing of ubiquitinated protein aggregates (Eskelinen, 2006; Isakson et al., 2013).

To test whether these changes in autophagy‐related proteins globally modified autophagy in fibroblasts as a function of donor age, we assessed differences in LC3‐I/II conversion in fibroblasts from young donors (<35 years old) relative to fibroblasts from old donors (>75 years old) using western blot analysis. In line with the proteomic data suggesting a rise in autophagy, the levels of LC3‐II relative to LC3‐I were significantly higher in older donors; GAPDH levels were assessed to monitor loading (Figure 3b, left). It was reported that p62/Sequestosome‐1 (SQSTM1) can directly interact with LC3 and GABA type A receptor‐associated protein (GABARAP) family members, thus facilitating the degradation of ubiquitinated proteins (Pankiv et al., 2007). During this process, p62 itself is degraded by autophagy and thus is often used as a marker to monitor autophagic flux (Bjorkoy et al., 2009). Notably, the levels of p62 were lower with age in our proteomic analysis (Figure 3b, right).

To ensure redox balance, cells are equipped with enzymatic antioxidants such as superoxide dismutase, glutaredoxins, and thioredoxins, as well as nonenzymatic antioxidants such as vitamin E, vitamin C, and glutathione (Birben et al., 2012). Among the multiple proteins capable of scavenging free radicals, we found that the levels of CuZn‐superoxide dismutase (CuZn‐SOD or SOD1), which catalyzes the dismutation of superoxide radicals to molecular oxygen and hydrogen peroxide (Zelko et al., 2002), were significantly higher with donor age (Figure 3c). In addition, glutaredoxin 1 (GLRX1), a member of the family of glutaredoxins, which play essential roles in protein homeostasis in response to stress (Feleciano et al., 2016), was significantly more abundant with older age in our proteomic analysis (Figure 3c). Furthermore, thioredoxin reductases, a family of selenium‐containing proteins which can catalyze the NADPH‐dependent reduction in the redox protein thioredoxins (Mustacich & Powis, 2000), were also higher with aging. Among them, thioredoxin reductase 1 (TRXR1) was found significantly elevated with age in our analysis (Figure 3c). Lastly, the levels of several glutathione S‐transferases, a family of enzymes that catalyze the conjugation of glutathione in the cytosol, mitochondria, and microsomal compartment (Hayes et al., 2005), were higher with age in our analysis. In particular, glutathione S‐transferase kappa 1 (GSTK1) and MGST1 were significantly more abundant (Figure 3c). It is worth noting that MGST1 was among the proteins most significantly overrepresented with age in this paradigm (Figure 1d).

Given that multiple antioxidant proteins and pathways were elevated with age, we investigated ROS levels in fibroblasts from donors of different ages by assessing H_2_DCFDA fluorescence using flow cytometry analysis. We detected lower ROS levels in young donors (<35 years old) compared with older donors (>75 years old) (Figure 3d), suggesting that the rise in antioxidant enzymes might be aimed at counteracting the increased ROS.

### 2.3 Pathways leading to ribosome biogenesis, DNA replication, and DNA repair tend to be suppressed in fibroblasts from older donors

**FIGURE 4:**
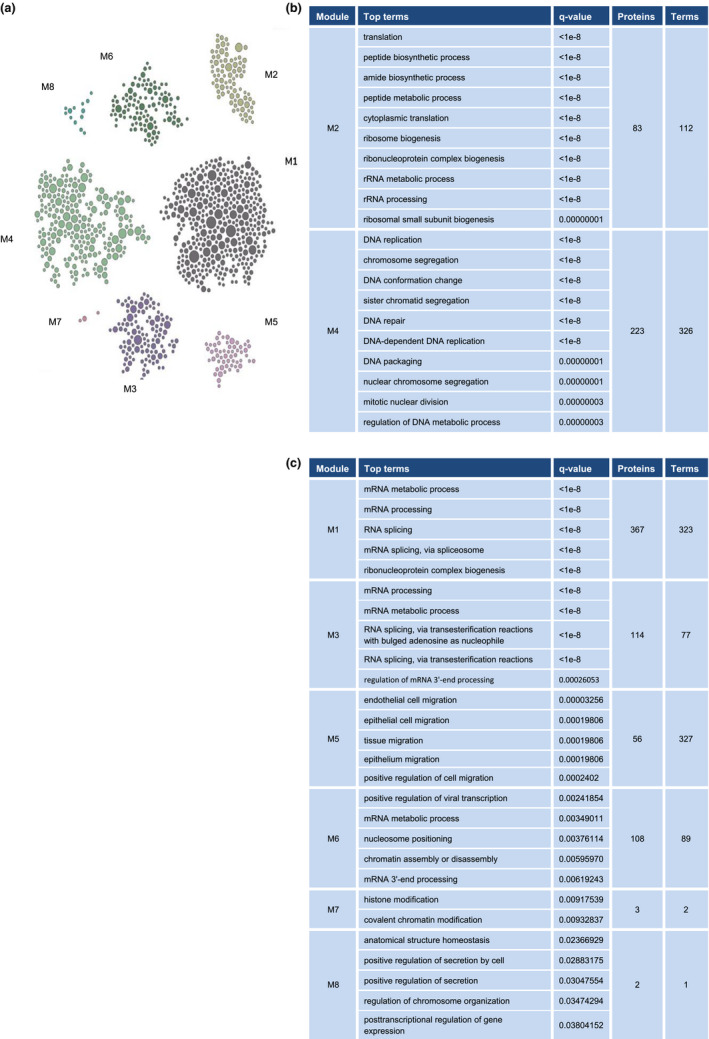
Pathways significantly underrepresented with age. (a) Modules significantly underrepresented with age. Each module consists of significantly decreased pathways (top term) with shared proteins. (b) We further investigated module 2 (M2), predominantly consisting of proteins involved in ribosome biogenesis and translation, and module 4 (M4), mainly consisting of proteins related to DNA replication and repair. (c) Remaining modules less represented with older age

Pathway enrichment analysis and age‐associated functional module analysis also identified several major functional modules that were lower with advancing age (Figure 4a). We focused on those pathway modules involved in ribosome biogenesis and DNA repair and replication, represented in modules 2 and 4, respectively (Figure 4b). Other prominently reduced pathways were related to general mRNA metabolic process, including mRNA splicing and regulation of 3’ end processing (Figure 4c).

**FIGURE 5:**
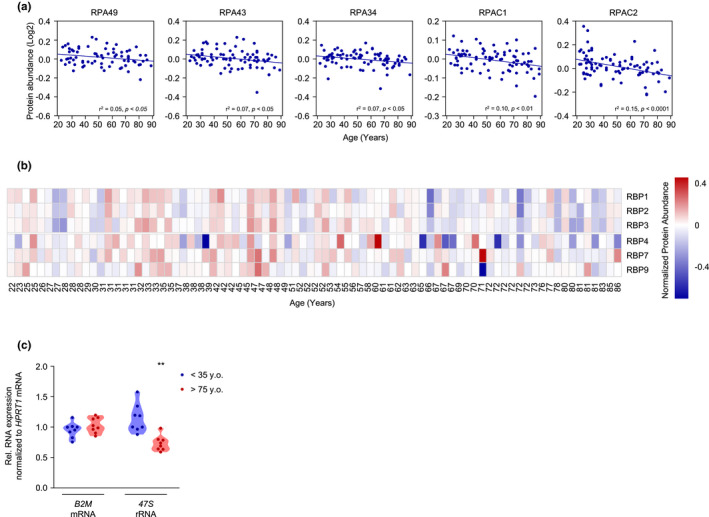
Lower levels of ribosomal proteins and ribosome biogenesis proteins. (a) Linear regression graphs of Pol I‐associated subunits significantly decreased with age. (b) Heatmap of Pol I‐specific subunits lower with older age. (c) Levels of *47S* rRNA (and *B2M* mRNA, as a control) in 8 donors <35 years old and 8 donors >75 years old, as quantified by RT‐qPCR analysis. Data were normalized to *HPRT1* mRNA, encoding a housekeeping protein. Pol I, RNA polymerase I; Pol II, RNA polymerase II. Significance was established using Student's *t* test. **p ≤ 0.01

There is increasing evidence that dysregulated ribosome biogenesis is associated with aging and age‐related diseases (Turi et al., 2019). Ribosome biogenesis is regulated through signaling via mechanistic target of rapamycin (mTOR) and the mTOR kinase complex (mTORC1), which in turn regulates all RNA polymerases (Pol I, Pol II, and Pol III) (Mayer & Grummt, 2006). Notably, Pol I transcribes *47S* rRNA, the precursor of *18S*, *5*.*8S*, and *28S* rRNAs (Henras et al., 2008), and thereby affects ribosome biogenesis. We found an age‐associated reduction in the levels of Pol I‐specific subunits RPA49 (PAF53), RPA43, and RPA34 (PAF49), as well as in two subunits shared by both Pol I and Pol III, RPAC1 and RPAC2 (Figure 5a) (Russell & Zomerdijk, 2006). Notably, the levels of prominent Pol II‐specific subunits RBP1, RBP2, RBP3, RBP4, RBP7, and RBP9 (Hahn, 2004) were also significantly lower with advancing donor age (Figure 5b). Given that several Pol I subunits decreased in our proteomic analysis, we tested whether the production of Pol I‐transcribed *47S* rRNA declined with age. As shown in Figure 5c, while *B2M* mRNA levels were unchanged, *47S* rRNA levels decreased in old donors compared with young donors, supporting the view that ribosome biogenesis was impaired in older‐donor fibroblasts.

**FIGURE 6:**
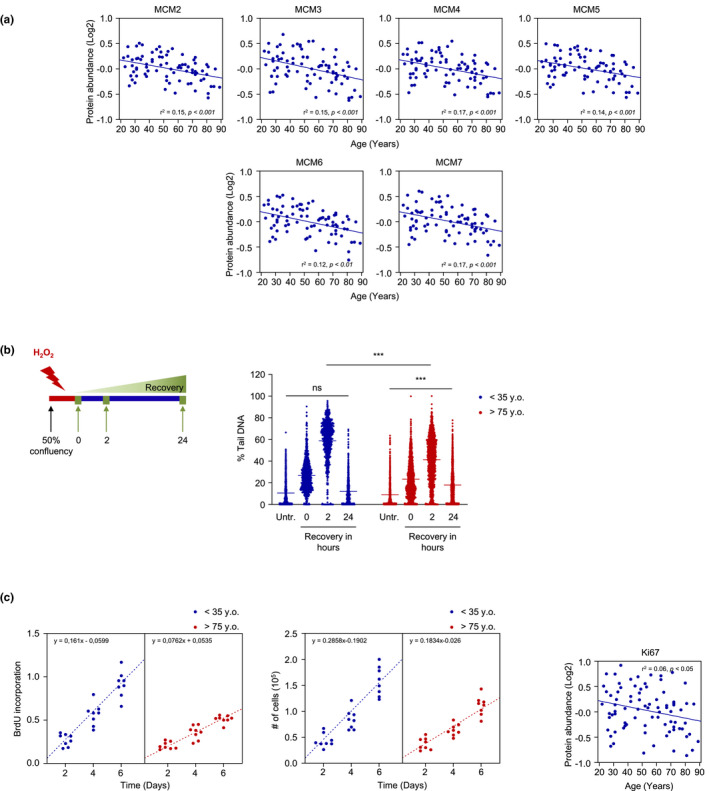
Lower levels of proteins implicated DNA metabolism with age, and assessment of the DNA replication and repair pathways. (a) Linear regression graphs of members of the MCM family associated with DNA replication and repair and significantly lower with older age. Donor ages are indicated at the bottom. (b) Schematic of the experiment measuring the DNA repair response in HDFs after H_2_O_2_ treatment (left) and the DNA repair response, as assessed using the comet assay (right). (c) BrdU incorporation (left) and number of cells (middle) were assessed in 8 donors <35 years old and 8 donors >75 years old up to day 6 (Materials and Methods). Linear regression graph of Ki67 levels based on the proteomic analysis (right). Significance was established using Student's *t* test. **p ≤ 0.01, ***p ≤ 0.001. MCM2‐7, minichromosome maintenance complex components 2–7; Ns, not significant

The second pathway showing the most marked decline with age implicated key proteins involved in DNA metabolism. Genomic instability and the accumulation of DNA damage are defining hallmarks of aging (Lopez‐Otin et al., 2013). Following DNA damage, cell proliferation is halted to allow cells time to repair (Bartek et al., 2004; Hartwell & Weinert, 1989). Among the proteins and enzymes involved in DNA replication and repair, members of minichromosome maintenance (MCM) complex family orchestrate the initiation and elongation steps in DNA replication, as well as the response to DNA damage (Bochman & Schwacha, 2009; Drissi et al., 2018). Six highly conserved proteins (MCM2‐7) form a hexameric complex that mediates the initiation and elongation steps of DNA replication in eukaryotes and interacts with DNA repair pathway proteins (Deegan & Diffley, 2016). As shown in Figure 6a, the levels of MCM2‐7 were lower with donor age in our analysis, supporting the link between impaired DNA metabolism with advanced age and levels of MCM proteins. Of note, MCM6 was among the most significantly lower proteins in older donors in our proteomic analysis (Figure 1d).

Given the importance of the MCM complex in DNA repair, we investigated the capacity of primary skin HDFs to repair hydrogen peroxide (H_2_O_2_)‐induced DNA damage using the comet assay (Materials and Methods). To test the ability to repair DNA, we treated HDFs with H_2_O_2_ for 2 h and assessed the recovery from DNA damage at 0, 2, and 24 h after H_2_O_2_ treatment (Figure 6b, schematic). Treatment with H_2_O_2_ elicited comparable levels of DNA damage in young and old donors as the % tail DNA was comparable at 0 h. Interestingly, after 2 h of recovery, DNA damage was significantly higher in young donors relative to old; however, the damage was fully repaired after 24 h in young, but not in older donors (Figure 6b). These observations might reflect the fact that the higher levels of multiple antioxidants proteins in older donors under basal conditions (Figure 3c) may have elicited stress resistance initially, but a reduced complement of DNA repair factors may have hampered subsequent restoration of DNA integrity (Figure 6b), as proposed earlier (Dues et al., 2019).

Given that the MCM2‐7 complex was underrepresented in our proteomic analysis, we investigated whether DNA replication was also impaired in skin fibroblasts derived from older donors. Analysis of BrdU incorporation, a measure of DNA replication, revealed higher activity in young donors (<35 years old) than in older donors (>75 years old), indicating that DNA replication was impaired with age (Figure 6c, left). In line with the differences in the BrdU assay, direct cell counting showed that fibroblasts from young donors proliferated faster than fibroblasts from older donors (Figure 6c, middle). Notably, the levels of the proliferation marker Ki67 were lower with age in our proteomic analysis, further supporting the results observed in BrdU incorporation assay and cell counting data (Figure 6c, right).

## 3 DISCUSSION

Toward the goal of elucidating the gene expression programs that control skin homeostasis during aging, we systematically cataloged changes in proteins expressed by primary fibroblasts isolated from skin biopsies of 82 persons ranging between 22 and 89 years old, from the GESTALT study of the NIA, a cohort in which we previously analyzed plasma and skeletal muscle (Tanaka et al., 2018; Tumasian et al., 2021; Ubaida‐Mohien et al., 2019). After deep quantitative analysis of the proteins differentially expressed as a function of donor age, we identified several major pathways in which these proteins were implicated. We focused on two pathways that were more represented with older age (autophagy and antioxidant defense) and two that were less represented with older age (ribosome biogenesis, and DNA replication and repair). We employed cell culture assays to ascertain experimentally if such pathways were differentially functional in fibroblasts from older compared with younger individuals. Our findings underscore several key processes that were more active and some that were less active in primary skin fibroblasts depending on the age of the donor and could be targeted therapeutically to preserve the functional integrity of aging skin.

### 3.1 Pathways overrepresented in primary skin fibroblasts from older donors

Autophagy is generally impaired with advanced age (Aman et al., 2021), and accumulating evidence suggests that enhanced autophagy may be linked to healthy aging and longevity, including reports that (i) mice overexpressing autophagy‐related protein ATG5 showed increased mean life span compared with wild‐type mice (Pyo et al., 2013); (ii) both caloric restriction and rapamycin, potent stimulators of autophagy, have been implicated in longevity (de Cabo et al., 2014; Wilkinson et al., 2012); and (iii) healthy centenarians expressed high levels of the autophagy protein Beclin‐1 in serum (Emanuele et al., 2014). Also, it was recently reported that autophagosomes accumulate in HDFs from healthy older women presumably as compensation to blockage of autophagic degradation (Tashiro et al., 2014), although it did not change significantly in HDFs from healthy older men as a function of age (Kim et al., 2018). In our study, we identified several autophagy‐related proteins that were more abundant in older donors (Figure 3a,b). Notably, autophagy was also predicted to be more active with advancing age in muscle biopsies of the same GESTALT cohort (Ubaida‐Mohien et al., 2019), which may further emphasize the importance of autophagy in healthy aging.

The antioxidant defense pathway was also prominently overrepresented in old‐donor fibroblasts. Despite earlier popularity of the free radical theory of aging (Harman, 1956), which posits that accumulation of ROS is a cause of aging, it is now widely recognized that the role of ROS in longevity is complex and remains incompletely understood. Both the levels and the sources of ROS are important for the effect on longevity (Beckman & Ames, 1998; Gladyshev, 2014). For example, genetic or pharmacological interventions leading to modest increases in ROS production extended *Caenorhabditis elegans* life span (Urban et al., 2017; Van Raamsdonk & Hekimi, 2009; Yang & Hekimi, 2010), and NDI1‐induced ROS accumulation extended the life span of *Drosophila melanogaster* (Scialo et al., 2016), although ablating prominent antioxidant enzymes did not seem to always affect mouse life span (Basisty et al., 2016; Huang et al., 2000; Perez et al., 2009; Schriner et al., 2005; Zhang et al., 2009).

There is strong evidence that mitochondrial function and integrity become impaired with aging and dysfunctional mitochondria may produce ROS levels that exceed the buffering capacity of the antioxidant system; aberrant redox states in turn promote cell cycle arrest and premature senescence (Hayflick, 1965; Stockl et al., 2006). Not only does oxidative phosphorylation induce premature senescence, but cellular senescence also affects mitochondrial respiration, predominantly through the dysregulation of glycolytic enzymes (Zwerschke et al., 2003). This vicious cycle of enhanced ROS accumulation and reduced antioxidant defense contributes to skin aging. In our proteomic analysis, we identified several antioxidant proteins preferentially elevated with age (Figure 3c). Surprisingly, some of the overrepresented antioxidant proteins were previously linked to the life span of model organisms. For instance, ablation of the *GLRX1* gene was previously associated with life span shortening and induction of cell senescence in *Saccharomyces cerevisiae* and in mammalian cell culture models, respectively (Liu et al., 2018; Yang et al., 2018); overexpression of TRXR1 extended the mean, but not the maximum, mouse life span (Perez et al., 2011); and MGST1 abrogation reduced the life span of *D*. *melanogaster* (Toba & Aigaki, 2000). Given that the nuclear factor erythroid 2‐related factor 2 (NRF2), which transcriptionally promotes the expression of multiple antioxidant proteins (Ma, 2013), is activated upon stress, the overall higher level of enzymatic antioxidants in our proteomic analysis is in line with the elevation of ROS production in older donors (Figure 3d). Whether these proteins affect human skin aging and might be therapeutic targets to improve skin aging warrants further investigation.

### 3.2 Pathways less active in primary skin fibroblasts from older donors

Studies in *C. elegans* and *S*. *cerevisiae* have highlighted the importance of ribosome biogenesis in life span. Genome‐wide RNA interference (RNAi) screens in *C. elegans* found that several prominent genes involved in ribosome biogenesis affected life span (Hamilton et al., 2005; Hansen et al., 2005). Similarly, the ribosomal proteins RPL10 and RPS6 as well as GCN4‐mediated depletion of the 60S ribosomal subunits can affect *S*. *cerevisiae* life span (Chiocchetti et al., 2007; Steffen et al., 2008). In addition, methylation of the *25S* rRNA modulated the life span of several model organisms (Schosserer et al., 2015), while genes related to ribosome function in HDFs decreased with age (Jung et al., 2015). Our proteomic analysis identified several Pol I‐ and Pol II‐specific subunits preferentially reduced with age (Figure 5a,b). Previous studies have reported the downregulation of Pol II‐specific subunits (Waldera‐Lupa et al., 2014), but little is known about the importance of Pol I in human aging and in particular in skin aging. Interestingly, it was recently reported that partial inhibition of Pol I may increase *D*. *melanogaster* life span (Martinez Corrales et al., 2020), a finding that deserves further study in human skin aging.

With respect to MCM2‐7 proteins, it is easy to envision that they may have a prevalent role in aging, given their impact during the initiation and elongation steps of DNA replication and during the DNA damage response. However, the role of MCM proteins in aging has not been studied thoroughly. It was previously reported that the levels of MCM2‐7 proteins were significantly lower in HDFs derived from healthy aged donors, and a role for MCM7 in proliferation was established by selective knockdown approaches (Dumit et al., 2014). Similarly, the levels of MCM2‐7 proteins were reported to decrease in replicative senescence (Suzuki et al., 2019). Notably, in both studies, MCM7 translocated to the cytosol with either increasing age or senescence, suggesting that besides steady‐state levels, the exit from the nucleus could represent a full loss‐of‐function for MCM7. In our proteomic analysis, the levels of MCM proteins were significantly lower with age (Figure 6a), potentially underscoring the importance of this protein family in DNA metabolism in chronological skin aging.

### 3.3 Study limitations and concluding remarks

Although cultured primary fibroblasts offer a valuable ex vivo model to identify, validate, and investigate specific proteins and pathways altered in persons of different ages, they do not faithfully recapitulate all traits of aging human skin. Fibroblasts are quiescent and are exposed to lower oxygen tension and different endogenous factors, but their ability to proliferate and respond to stress may be an important component of skin aging. Many earlier studies used ex vivo primary fibroblasts to identify molecules affecting the progression of diseases, highlighting the value of this cell model (e.g., Katarkar et al., 2020; Zehender et al., 2021). Future studies in vivo will be needed to complement the collective results gained from cell culture. As technologies advance, single‐cell proteomics (Marx, 2019) and spatial proteomics are expected to enable the identification of proteins expressed by fibroblasts in the intact skin with sufficient depth and precision. This information will be particularly valuable if combined with future spatial metabolomic analysis to provide insight into the function of protein pathways. The current work paves the way for future methodologies that can investigate fibroblast function in situ.

Throughout the years that it took to collect fibroblasts from all skin biopsies, we did not observe obvious differences in the levels of the senescence marker β‐galactosidase activity in older donors, suggesting that the continued proliferation of fibroblasts reflects their persistent ability to maintain homeostatic protection of our skin. Thus, it was somewhat unexpected to identify, upon deep proteomic and pathway analyses, several key processes that did become altered with advancing donor age. Here, we focused on the most robustly altered pathways and found that several hallmarks of skin aging were retained in isolated and cultured dermal fibroblasts, such as altered protein turnover, DNA replication, and antioxidant defense. However, the impact of many individual proteins and additional protein pathways await future research in aged skin.

We recognize that the strict donor health criteria precluded the inclusion of very old individuals (older than 89) in the study, so the proteomes of centenarians, reported in other studies (Miura et al., 2011; Santos‐Lozano et al., 2020; Sebastiani et al., 2021), could not be analyzed here. Nonetheless, with the wide age range of human subjects tested, the stringent inclusion criteria, and the state‐of‐the‐art mass spectrometry analysis performed, our study identified both previously reported and unreported proteins altering with age. Our work expands upon recent studies of the proteome of skin fibroblasts from a focused group of donors (Dyring‐Andersen et al., 2020) to identify several processes changing significantly with age. One important observation deserves to be noted. While we focused on four altered pathways (autophagy, ROS scavenging, ribosome biogenesis, and DNA replication/repair), the individual proteins were not drastically altered in general across all individuals within an age group. Instead, the altered phenotypes reflected the *joint function* of these proteins in specific pathways. Here, we provide a consolidated skin fibroblast proteome analysis of proteins and pathways that change with age. We thus propose that the altered molecular processes and pathways, rather than the proteins themselves, are promising targets to decelerate skin aging.

## 4 MATERIALS AND METHODS

### 4.1 Skin biopsies and culture of skin fibroblasts

Punch skin biopsies (4‐mm^2^) were obtained from the inner axilla, typically a photoprotected region, of 82 healthy GESTALT participants following a stringent clinical protocol that minimized the risk of infections and side effects, as described (Tanaka et al., 2018; Ubaida‐Mohien et al., 2019). Briefly, participants were enrolled in GESTALT if they were free of major diseases, were not taking drugs except a single monotherapy for hypertension, had no physical or cognitive impairments, did not train professionally, and had a body mass index (BMI) less than 30 kg/m^2^. The inclusion criteria were assessed during a 6‐hour clinical evaluation at the Clinical Research Unit of the NIA IRP based on medical history, physical exams, and blood tests interpreted by a trained nurse practitioner (Schrack et al., 2014). Skin biopsies were then minced into smaller pieces and distributed into 3 wells of collagen‐coated, 6‐well plates. The minced biopsies were incubated in DMEM (Gibco) supplemented with 20% fetal bovine serum (Gibco), 1% penicillin‐streptomycin (Gibco), and 1% non‐essential amino acids (Gibco) at 37°C in a humidified atmosphere for 2 weeks; then in DMEM supplemented with 10% FBS, 1% penicillin‐streptomycin, and 1% non‐essential amino acids for 1–2 weeks until confluent. The cultures were then expanded to three 100‐mm tissue culture plates, grown until confluency, and the established human diploid fibroblast (HDF) cell lines were frozen. After thawing, HDFs were cultured in DMEM supplemented with 10% FBS, 1% penicillin‐streptomycin, and 1% non‐essential amino acids at 37°C in a humidified atmosphere until they reached confluency and were used within 4 passages. The GESTALT protocol is approved by the Intramural Research Program of the US National Institute on Aging and the Institutional Review Board of the National Institute of Environmental Health Sciences. All participants provided written informed consent at every visit.

### 4.2 Sample collection, preparation, and protein extraction

The 82 participants were grouped as follows: 20–34 years old, *n* = 20; 35–49 years old, *n* = 17; 50–64 years old, *n* = 18; 65–79 years old, *n* = 18; 80+ years old, *n* = 9. In 100‐mm tissue culture plates, early‐passage HDFs were cultured as described above; cells were then washed twice with 1× phosphate‐buffered saline (PBS), scraped off the plates in 10 ml PBS, and centrifuged at 300 *g* for 5 min at 4°C. After removing the supernatant, cells were lysed in lysis buffer [100 mM Tris (pH 7.6) 100 mM Dithiothreitol (DTT), 150 mM NaCl, 4% Sodium Dodecyl Sulfate (SDS), and 1% Triton X‐114] supplemented with 5% v/v protease inhibitor cocktail (Sigma‐Aldrich) for 10 min on ice. After a 10‐min centrifugation at 10,000 *g*, protein lysates were collected and then denatured by incubating lysates at 95°C for 15 min; the lysates were then spun down for 10 min at 10,000 *g*, and the supernatants were stored at −80°C in 100‐μl aliquots. Protein concentration was assessed using a 2‐D Quant Kit (GE Healthcare Life Sciences); detergents and lipids were removed using a methanol/chloroform extraction protocol (Wessel & Flugge, 1984). From each sample, 100‐μg protein aliquots were resuspended in 30 μl of urea buffer (8 M urea, 2 M thiourea, 150 mM NaCl), reduced with 50 mM DTT and alkylated with 100 mM iodoacetamide each for 1 h at 36°C. They were then diluted 12 times with 55 mM ammonium bicarbonate containing 0.1% of proteasemax trypsin enhancer (Promega) and digested for 18 h at 36°C using a 1:50 (w/w) trypsin/LysC mixture (Promega). Peptides were desalted, speed vacuum‐dried, and stored at −80°C.

Tandem mass tag (TMT) labeling was used to perform quantitative proteomics according to the manufacturer's instructions (TMT10plex, Thermo Fisher). Each TMT labeling reaction contained 10 labels to be multiplexed in a single mass spectrometry (MS) run. We used 9 different TMT sets, and each set included one reference sample throughout the entire study to allow comparisons among different MS runs. To avoid bias, donor IDs were blinded, and TMT channels were randomized between the runs. Each sample was spiked with 200 fmol of bacterial β‐galactosidase digest (SCIEX) prior to TMT labeling to control for labeling efficiency and instrument performance. Labeled peptides were pooled and fractionated using standard basic reversed‐phase fractionation method.

### 4.3 High‐pH RPLC fractionation and concatenation strategy

High‐pH RPLC fractionation was performed on Agilent 1260 bio‐inert HPLC system using 2.0‐mm × 5‐mm XBridge BEH Shield RP18 XP VanGuard cartridge and 2.1‐mm × 250‐mm XBridge Peptide BEH C18 column (Waters). The solvent was composed of 10 mM ammonium formate (pH 10) and 10 mM ammonium formate and 90% ACN (pH 10) as mobile phases A and B, respectively (Wang et al., 2011). TMT‐labeled peptides prepared from the HDFs were separated using a linear organic gradient (5%–50% B, 80 min); 75 fractions were collected during each liquid chromatography (LC) run at 1‐min interval each and then pooled into 15 fractions. Pooled fractions were speed vacuum‐dried, desalted, and stored at −80°C until liquid chromatography–mass spectrometry (LC‐MS/MS) analysis.

### 4.4 LC‐MS/MS analyses

Purified peptide fractions from HDFs were analyzed using UltiMate 3000 Nano LC Systems coupled to the Orbitrap Fusion™ Lumos™ Mass Spectrometer (Thermo Scientific). Each fraction was separated on a 50‐cm capillary column with 150 μm ID using a linear organic gradient with 550 nl/min flow rate. Mobile phases A and B consisted of 0.1% formic acid in water and 0.1% formic acid in acetonitrile, respectively. Tandem mass spectra were obtained with a heated capillary temperature of 320°C and spray voltage set to 2.5 kV. Full MS1 spectra were acquired from 300 to 1,500 m/z at 120,000 resolution and 50 msec maximum accumulation time with automatic gain control set to 2 × 10^6^. Dd‐MS2 spectra were acquired using dynamic m/z range with fixed first mass of 100 m/z. MS/MS spectra were resolved to 50,000 with 100 msec of maximum accumulation time and AGC target set to 1 × 10^5^. The fifteen most abundant ions were selected for fragmentation using 35% normalized high collision energy. A dynamic exclusion time of 70 s was used to discriminate against the previously analyzed ions. The mass spectrometry proteomics data are deposited at the MassIVE site https://massive.ucsd.edu/ProteoSAFe/static/massive.jsp with the dataset identifier MSV000088401.

### 4.5 Bioinformatic analysis of proteomic data

The raw data generated from each sample fraction were converted to mascot generic format (mgf) using MSConvert software (ProteoWizard 3.0.6002) and then searched with Mascot 2.4.1 and X!Tandem CYCLONE (2010.12.01.1) using the SwissProt Human sequences from Uniprot (Version Year 2021, 20,300 sequences, appended with 115 contaminants) database. For searching, the engine was set with the following parameters: TMT10plex lysine and N‐terminus as fixed modifications and variable modifications of carbamidomethyl cysteine, deamidation of asparagine and glutamate, carbamylation of lysine and N‐terminus and oxidized methionine. A peptide mass tolerance of 20 ppm, 0.08 Da, and two missed cleavages were allowed for precursor and fragment ions according to the known mass accuracy of the instrument. Mascot and X!Tandem search engine results were analyzed in Scaffold 11.2 (Proteome Software, Inc.). The isotopic purity of the TMT channels was corrected according to the TMT kit. Peptide and protein probability was calculated by PeptideProphet and ProteinProphet probability models, respectively (Keller et al., 2002; Nesvizhskii et al., 2003). Proteins were filtered at thresholds of 0.01% peptide false discovery rate (FDR), 1% protein FDR, and requiring a minimum of 1 unique peptide for protein identification. Unique and single peptides were included only if the identification was confirmed by more than one search engine and quantified in multiple participants, as described (Ubaida‐Mohien et al., 2019). The log2‐transformed spectral abundance was normalized by median subtraction from all reporter ion intensity spectra belonging to a protein across all channels. All TMT sets were normalized to each other by a global normalization factor calculated from the average of all channels’ median divided by the median of each channel spectra quantified. Protein sample loading effects from sample preparations were corrected by median polishing, that is, subtracting the channel median from the relative abundance estimate across all channels to have a zero median. Relative protein abundance was estimated by the median of all peptides for a protein combined together (Herbrich et al., 2013; Kammers et al., 2015).

### 4.6 Western blot analysis

To assess autophagy, 10^5^ cells were seeded per well and grown to 70% confluency. Cells were then lysed in RIPA buffer (10 mM Tris‐HCl, 150 mM NaCl, 1 mM EDTA, 1% NP‐40, 0.1% SDS) supplemented with 1× protease and phosphatase inhibitor cocktail (Thermo Scientific) and processed as described (Tsitsipatis et al., 2021). Incubation with antibodies recognizing LC3‐I/II and GAPDH (Supplementary File S2, *Antibodies*), diluted in 1% milk in TBS‐Tween, was carried out for 16 h at 4°C; membranes were then washed with TBS‐Tween for 5 min and incubated for 1 h with secondary antibodies conjugated with horseradish peroxidase (KwikQuant) diluted in 1% milk in TBS‐Tween and then washed with TBS‐Tween for 5 min, at 25°C. Membranes were developed using Enhanced Chemiluminescence (ECL), and digitized images were captured using KwikQuant Imager (Kindle Biosciences).

### 4.7 Cell counting and BrdU incorporation

Cell proliferation was assessed as described (Tsitsipatis et al., 2021). Briefly, 2 × 10^4^ cells were plated per well and proliferation rates were assessed using both cell counting and BrdU incorporation up to day 6. For cell counting, cells were harvested and washed once with 1× PBS (Gibco), and total cells were counted on an automatic cell counter (Bio‐Rad). For BrdU incorporation, BrdU Cell Proliferation Assay (Cell Signaling Technology) was employed following the manufacturer's instructions. Briefly, BrdU was added one day after seeding and the incorporation was detected for up to day 6 by reading at 450 nm on a GloMax Explorer plate reader (Promega).

### 4.8 Comet assay

To assess the ability of the HDFs to repair DNA damage, 10^4^ cells were plated per well and grown to 50% confluency. Cells were then treated with 50 μM of hydrogen peroxide (H_2_O_2_, Sigma‐Aldrich) for 2 h followed by recovery for 2 h or 24 h (Ko et al., 2012). Following the recovery time, cells were harvested and washed twice with 1× PBS, and total live and dead cells were counted on an automatic cell counter after staining with 0.4% Trypan Blue (Gibco). In addition, 5 × 10^4^ cells were plated per well and grown until confluency to assess DNA damage repair under basal conditions. The capacity of the cells to repair DNA damage was assessed using Trevigen Comet Assay kits as described (Beerman et al., 2014). Slides were visualized on an Applied Precision DeltaVision Microscope System and captured using the Olympus IX‐71 10× objective lens and FITC filter set. Images were collected in a 11 × 11 array for each well, with each image composed of three optical sections separated by 1.6 μm.

### 4.9 Reactive oxygen species accumulation

To assess the accumulation of reactive oxygen species (ROS), 10^5^ cells were seeded per well and grown until 70% confluency. Cells were then harvested, washed twice with 1× Dulbecco's PBS (DPBS, Gibco), and incubated with 1 μΜ of 2’,7'‐dichlorodihydrofluorescein diacetate (H_2_DCFDA; Thermo Fisher Scientific) for 30 min at 25°C in the dark. Cells were then washed once with DPBS and analyzed with FACS Aria FUSION (BD Bioscience) in the dark. FACS Diva software (BD Bioscience) was employed to assess FITC mean intensity.

### 4.10 RNA isolation, reverse transcription (RT), and quantitative (q)PCR analysis

To isolate total RNA, 10^5^ cells were seeded per well and grown until 70% confluency. Cells were then harvested and washed once with 1× PBS, and total RNA was isolated using the Direct‐zol^™^ RNA MiniPrep kit (Zymo Research) following the manufacturer's instructions. For reverse transcription (RT) followed by quantitative PCR (qPCR) analysis, 1 μg of total RNA was used. For qPCR analysis, 0.1 µl cDNA was used with 250 nM of primers (Supplementary File S2, *Primer pairs*) and KAPA SYBR^®^ FAST qPCR Kits (KAPA Biosystems). RT‐qPCR analysis was carried out on a QuantStudio 5 Real‐Time PCR System (Thermo Fisher Scientific) with a cycle setup of 3 min at 95°C, 40 cycles of 5 s at 95°C, and 20 s at 60°C. Relative RNA levels were calculated after normalizing to *HPRT1* mRNA using the 2^−ΔΔCt^ method.

### 4.11 Statistical and bioinformatic analysis

A multiple linear regression model was used to examine the effect of age on each protein after adjusting for gender, race, and body mass index (BMI). The regression model was performed using R 3.6.1 (R Core Team, 2016) with the lm() R linear regression function. Proteins with a negative beta coefficient were considered to be negatively associated with age and the proteins with positive beta coefficient as positively associated with age. The size of the beta coefficient for age was used to quantify the effect of age (per year) on each protein independent of covariates, and the significance of the association from the regression model was determined with *p*‐values. Any protein with *p* < 0.05 was considered to be significant and reported in the main text as a protein associated with age. Multiple testing corrections of the *p*‐values were performed using the Benjamini–Hochberg method in R and reported in the supplemental tables. Protein annotations were performed using GeneOntology, Uniprot keyword, and manual curation. Pathway enrichment analysis was performed using STRING functional enrichment tool with Reactome and KEGG background database (Szklarczyk et al., 2021). HumanBase functional protein module analysis was used for identifying cohesive gene clusters and for representing local gene network neighborhood from age‐associated genes (Greene et al., 2015; Ju et al., 2013). Heatmaps and hierarchical cluster analysis were performed using R the non‐linear minimization package. R packages limma, qvalue, ggplot2, and pheatmap were used for statistical analysis and plotting figures. Quantitative data are represented as the means ± SD and compared statistically by unpaired Student's *t* test, using GraphPad Prism (9.0). A *p*‐value of < 0.05 was considered statistically significant and indicated in the figures as **p < 0.01. Volcano plot, heatmaps, linear regression, and violin graphs were generated using GraphPad Prism (9.0).

## CONFLICT OF INTEREST

The authors declare that they have no conflicts of interest.

## AUTHOR CONTRIBUTIONS

DT, LF, and MG conceived the study; DT, JLM, RM, and MG designed experiments; DT, JLM, CUM, AL, HY, AK, CHS, ABH, EJ, JHY, RM, CD, YL, and IB performed and analyzed experiments; MK, CYC, RM, and XY contributed intellectually and provided technical support; CHC, ACK, LZ, JD, and LF collected the human biopsies; DT, JLM, CUM, AL, LF, and MG wrote the manuscript.

## Supporting information

Supplementary File S1

Supplementary File S2

## Data Availability

The mass spectrometry proteomics data have been deposited at MassIVE with the dataset identifier MSV000088401 (https://massive.ucsd.edu/ProteoSAFe/static/massive.jsp, ftp://MSV000088401@massive.ucsd.edu).
